# Effect of acupuncture on the energy metabolism of dogs with intervertebral disk disease and cervical disk herniation: A pilot study

**DOI:** 10.1007/s11259-022-10051-4

**Published:** 2022-12-19

**Authors:** Megumi Sawamura, Toshiro Arai, Koh Kawasumi

**Affiliations:** 1 East Chiba Animal Medical Center, Chiba, Japan; 2 School of Veterinary Medicine, Nippon Veterinary and Life Science University, 1-7-1 Kyonancho, Musashino, Tokyo 180-8602 Japan

**Keywords:** Acupuncture, Dog, Energy metabolism

## Abstract

Thirteen dogs with intervertebral disk disease (IVDD) and 3 dogs with cervical disk herniation (CVDH) were examined to determine the effects of acupuncture on energy metabolism. Acupuncture points GV14, GV20-1, BL18, BL23, BL26, GB30, and ST36 were selected for IVDD, while GV14, GV20-1, GB20, and BL23 were selected for CVDH. All dogs except no.13 did not receive medication during acupuncture treatment. Acupuncture effects were evaluated based on the IVDD/CVDH evaluation scales in Oji 2015 and Tanaka and Nakayama 2015. Blood samples were taken before and 30 min after acupuncture treatment. Pyruvate and lactate concentrations, lactate dehydrogenase (LDH) and malate dehydrogenase (MDH) activity, the MDH/LDH ratio (M/L ratio), and LDH isozyme electrophoretic patterns served as energy metabolism markers. In IVDD/CVDH dogs that showed improvements, plasma pyruvate concentrations significantly decreased, the M/L ratio increased, and the plasma LDH isozyme pattern changed from predominantly LDH5 to predominantly LDH1. These data suggest that local redox potential is improved and energy metabolism is increased in dogs with IVDD/CVDH after acupuncture treatment. Acupuncture treatments may activate the citric acid cycle and increase ATP production, followed by improvement of the disease. Future studies with a large sample size are needed to clarify this hypothesis.

## Introduction

Intervertebral disc disease (IVDD) causes canine gait disorders (Jeffery et al. 2013), and its prevalence increases with age (Smolders et al. 2013). Canine IVDD, including cervical disk herniation (CVDH), is more common in chondrodystrophic (CD) breeds such as Miniature Dachshunds, French Bulldogs, Welsh Corgis, Toy Poodles, Pekingese, and Shih Tzus. These CD breeds have genetic predisposition to develop IVDD/CDVH (Parker et al. 2009). CVDH accounts for 15% of all IVDD in dogs and the cervical spine (specifically the C2-3 vertebrae) is likely to suffer CVDH (Fry et al. 1991). Although IVDD/CDVH is generally treated with steroids, anti-inflammatory medication and surgery, many acupuncture treatments for canine IVDD/CDVH have been reported (Han et al. 2012; Liu et al. 2016; Roynard et al. 2018; Wright 2019). Generally, placing needles in acupuncture points is thought to not only enhance blood circulation but also stimulate the nervous systems and increase the release of anti-inflammatory and pain substances. Electroacupuncture involves continuous stimulation of the surrounding muscles with a low-frequency pulse and that induces muscle contraction. In Japan, electroacupuncture is popular due to a lack of complications due to treatment.

We applied acupuncture treatment to 106 dogs with IVDD/CVDH at Higashi Chiba Animal Medical Center (Togane City, Chiba) and the Animal Integrative Medical Center (Chiba City, Chiba), between February and October 2019. In the present study, we selected 16 dogs from this pool to exclude potential confound, such as use of other medication, supplements or kampo medicine. Then, to investigate the effects of acupuncture on energy metabolism in dogs with IVDD/CDVH, plasma pyruvate and lactate concentrations, malate dehydrogenase (MDH) and lactate dehydrogenase (LDH) activity, the MDH/LDH (M/L) ratio, and LDH isozyme electrophoretic patterns were examined. The plasma M/L ratio is one of the useful indicators of oxidative metabolism and that an increase in the M/L ratio suggests increased ATP production via the citric acid cycle (Li et al. 2012). To our knowledge, this is the first report about the effects of acupuncture on energy metabolism in dogs with IVDD /CVDH.

## Materials and methods

### Animals

We investigated the effects of acupuncture therapy on 16 dogs with IVDD. The included dogs ranged from 3–16 years of age, and represented the following breeds: Miniature Dachshund (8), Norfolk Terrier (2), Chihuahua (1), Miniature Schnauzer (1), French Bulldog (1), Maltese (1), Jack Russell Terrier (1), and mixed (1). No dogs (except no.13, which was given prednisolone and ursodeoxycholic acid for liver cancer), took any medications during the study period.

Before treatment, we obtained written informed consent from the owners of all the dogs. Ethic approval for this study was obtained from Higashi Chiba Animal Medical Center (Togane City, Chiba) (R2-1). This study was conducted following best veterinary practices.

### Acupuncture

In this study, 2 acupuncture treatment methods, electroacupuncture and needle acupuncture, were applied based upon traditional Chinese and Japanese veterinary medicine (Sawamura 2019). In the electroacupuncture methods, the acupuncture needles were placed in acupoints and continuously emitted electrical stimulation powered by an electric instrument (Lasper Ace, Kanaken Co., Ltd. Kanagawa) at low (3–50 Hz) frequencies for 20 min. In the needle acupuncture method, needles were placed in the acupuncture points for 15–20 min without electrostimulation. All patients received electroacupuncture or needle acupuncture once a week for 2 weeks. Six dogs (nos.1, 5, 6, 8, 9, and12) received electroacupuncture and 10 dogs (nos.2, 3, 4, 7, 10, 11,13,14,15, and 16) received needle acupuncture.

Once improvement was observed, the treatment frequency was reduced to once a month. Dogs with more severe IVDD/CVDH, as indicated by higher grades (IVDD G3 ~ 5, CVDH G3), received more acupuncture treatments; dogs with less severe IVDD/CVD, as indicated by lower grades (IVDD and CVDH G1 ~ 2) received fewer acupuncture treatments. All patients were diagnosed after 2 months, although some showed improvement in disease grade after 1 week. The acupuncture needles used in this study were as follows: 1) Seirin D-Type no.1. (φ0.16) × 15 mm (SEIRIN Corporation, Shizuoka, Japan), 2) D-Type no.3 (φ0.20) × 15 mm (SEIRIN Corporation, Shizuoka, Japan), 3) Kanaken Disposable Acupuncture φ0.16 × 30 mm, φ0.18 × 30 mm (Kanaken Co., Ltd. Kanagawa, Japan) and 4) Kanaken Disposable Acupuncture φ0.20 × 30 mm needles (Kanaken Co., Ltd. Kanagawa, Japan).

Acupuncture points GV14, GV20-1, BL18, BL23, BL26, GB30, and ST36 were selected for IVDD, while GV14, GV20-1, GB20, and BL23 were selected for CVDH (Sawamura 2019). Acupuncture effects were evaluated as improvement on the IVDD and CVDH grading scales (Oji 2015, Tanaka and Nakayama 2015). Cervical spinal cord disorders are classified as follows: grade 1, neck pain without neurological abnormalities; grade 2, gait disturbances and neurological abnormalities; and grade 3, additional difficulty standing and walking as well as neurological abnormalities (Oji 2015). Thoracolumbar spinal cord disorders are classified as follows: grade1 no spinal cord dysfunction but back pain; grade 2 mild paresis of the hind limbs; grade 3, severe paresis of the hind limbs; grade 4, hindlimb paralysis and deep pain sensation; and grade 5, paralysis of the hind limbs and no deep pain sensation (Tanaka and Nakayama 2015). The effect of acupuncture was evaluated over 2 months after treatment except for nos.14 and 15. The owners of these dogs stopped taking their pets to the hospital within a month after the end of treatment.

### Blood sampling and assays of metabolites and enzymes

Blood samples (2.5 mL) were collected from the jugular vein with heparin used as an anticoagulant before and after acupuncture treatment, which lasted approximately 30 min. After centrifugation at 1,200 rpm for 5 min at 4℃, plasma was obtained and stored at -40℃ until further analysis.

### Measurements of plasma metabolites concentrations and enzyme activity

Plasma pyruvate concentrations were measured by a previously described method (Czok and Lamprecht 1974) and lactate concentrations were measured with a commercial kit (Lactate Assay Kit-WST, Dojindo, Tokyo, Japan). Plasma LDH (Kaloustian et al. 1969) and MDH (Bergmeyer and Bernet 1974) activity were measured by previously described methods. The plasma M/L ratio was calculated as MDH activity divided by LDH activity. Plasma LDH isozyme patterns were detected by the biphasic agarose gel electrophoresis utilizing commercial Quick LD gels (Helena Laboratories, Saitama, Japan) (Hirakawa et al. 2012). The LDH fraction was assessed and analyzed using Quick Scan (Helena Laboratories, Saitama, Japan).

These measurements were carried out by one person in the Laboratory of Veterinary Biochemistry, Nippon Veterinary and Life Science University.

### Statistical analysis

Results are presented as the means ± standard deviations (SDs). Groups were compared with a paired-t tests. The significance level was set at P < 0.05. All analyses were performed using Microsoft Excel software.

## Results

In this study, electroacupuncture or needle acupuncture treatment were applied to 16 dogs. No concomitant treatments were administered (Table 1).

**Table 1:** Characteristics of the 16 dogs examined in this study

	Breed	Age	Gender	Condition of disease	Grade of IVDD/CVDH	
Group with grade improvement					Before treatment	After treatment
1	Norfolk Terrier	11	castrated	IVDD	G3	G0
2	M.dackshund	10	castrated	IVDD	G2	G0
3	M.dackshund	10.1	castrated	IVDD	G2	G0
4	Chihuahua	3.4	non spayed	CVDH	G3	G2
5	M.dackshund	12.4	non spayed	CVDH	G3	G0
6	M.dackshund	14.1	non castrated	IVDD, Medial patella luxation	G5	G2
7	M.shunauzer	15.4	spayed	IVDD, Degenerative spondylosis, Cauda equina syndrome	G2	G0
8	Norfolk Terrier	11.1	castrated	IVDD	G2	G0
9	French bulldog	9.2	spayed	IVDD, Canine atopic dermatitis	G2	G0
10	Maltese	10.5	spayed	CVDH	G3	G0
Group without grade improvement						
11	M.dackshund	16.11	spayed	IVDD,Hypothyroidism, Liver dysfunction → death due to old age	G3	death
12	Mixed	15	castrated	IVDD → death due to Myeclomalacia	G5	death
13	Jack russell terrier	14.3	castrated	IVDD, Liver tumor, Kidney failure → death due to cancer	G2	death
14	M.dackshund	15	spayed	IVDD, Liver dysfunction, Spondylosis → no improvement whithin 1 month hospitalization	G2	not allowed
15	M.dackshund	4.3	spayed	IVDD Liver dysfunction, Medial patella luxation	G2	not allowed
16	M.dackshund	16.1	castrated	IVDD Liver dysfunction, Torticollis, Gait disturbance due to Dementia	G2	G2

Abbreviations: IVDD, intervertebral disk disease; CVDH, cervical disk herniation

Acupuncture points: GV14, GV20-1, BL26, and ST36 for IVDD; GV14, GV20-1, GB 20, and BL23 for CVDH

The effect of acupuncture was evaluated 2 months after treatment, except for nos.14 and 15 (these dogs

could not be evaluated because their owners stopped bringing them to the hospitals during the experiment)

We found that acupuncture altered energy metabolism in dogs with IVDD/CVDH. As shown in Table 2, dogs with CVDH (G2 and 3) or IVDD (G2,3, and -5) at base line showed improvements. The dogs without improvement had similar severity of IVDD (G2,3 and 5) as well as comorbid disorders, such as hypothyroidism, liver dysfunction, kidney failure, spondylosis, medial patella luxation, torticollis, or dementia.

**Table 2:** Changes in plasma metabolites concentrations and enzyme activity in dogs with IVDD/CVDH before and after acupuncture treatment

		Group with grade improvement		Group without grade improvement	
		pre (n = 10)	post (n = 10)	pre (n = 6)	post (n = 6)
Pyruvate	(mmol L^−1^)	0.17 ± 0.08	0.10 ± 0.03*	0.21 ± 0.26	0.20 ± 0.12
Lactate	(mmol L^−1^)	22.98 ± 24.41	28.04 ± 28.62	6.04 ± 10.97	8.77 ± 17.77
LDH	(IU L^−1^)	67.06 ± 24.81	58.65 ± 22.10	99.02 ± 59.06	141.09 ± 126.43
MDH	(IU L^−1^)	55.69 ± 37.76	64.94 ± 42.56	61.30 ± 49.23	68.02 ± 64.50
M/L ratio		0.93 ± 0.67	1.17 ± 0.74	0.61 ± 0.28	0.60 ± 0.55
		pre (n = 7)	post (n = 7)	pre (n = 4)	post (n = 4)
LDH1	(%)	17.40 ± 10.52	31.84 ± 20.51	6.95 ± 3.73	11.58 ± 12.60
LDH5	(%)	65.07 ± 13.01	38.83 ± 14.15	69.18 ± 11.51	65.50 ± 12.96

Blood samples were collected before and after 30 min of acupuncture treatment during first session

Data are expressed as the means ± standard deviations (SDs)

^*^ Indicates significant differences (P < 0.05) from the pretreatment value according to a paired-t test

As shown in Table 2, plasma pyruvate concentrations significantly decreased after acupuncture treatment (from 0.17 ± 0.08 mM to 0.10 ± 0.03 mM) in dogs with IVDD/CVDH that improved after treatment. Plasma LDH activity decreased from 67.06 ± 24.81 IU L^−1^ to 58.65 ± 22.10 IU L^−1^, while plasma MDH activity increased from 55.69 ± 37.76 IU L^−1^to 64.94 ± 42.56 IU L^−1^. After acupuncture treatment, the plasma M/L ratio increased from 0.93 ± 0.67 to 1.17 ± 0.74 although this increase was not significant.

Plasma lactate concentrations did not significantly change. In the dogs that showed improvement, plasma pyruvate significantly decreased, while lactate concentrations and LDH and MDH activity did not change significantly after acupuncture treatment.

**Fig. 1:**
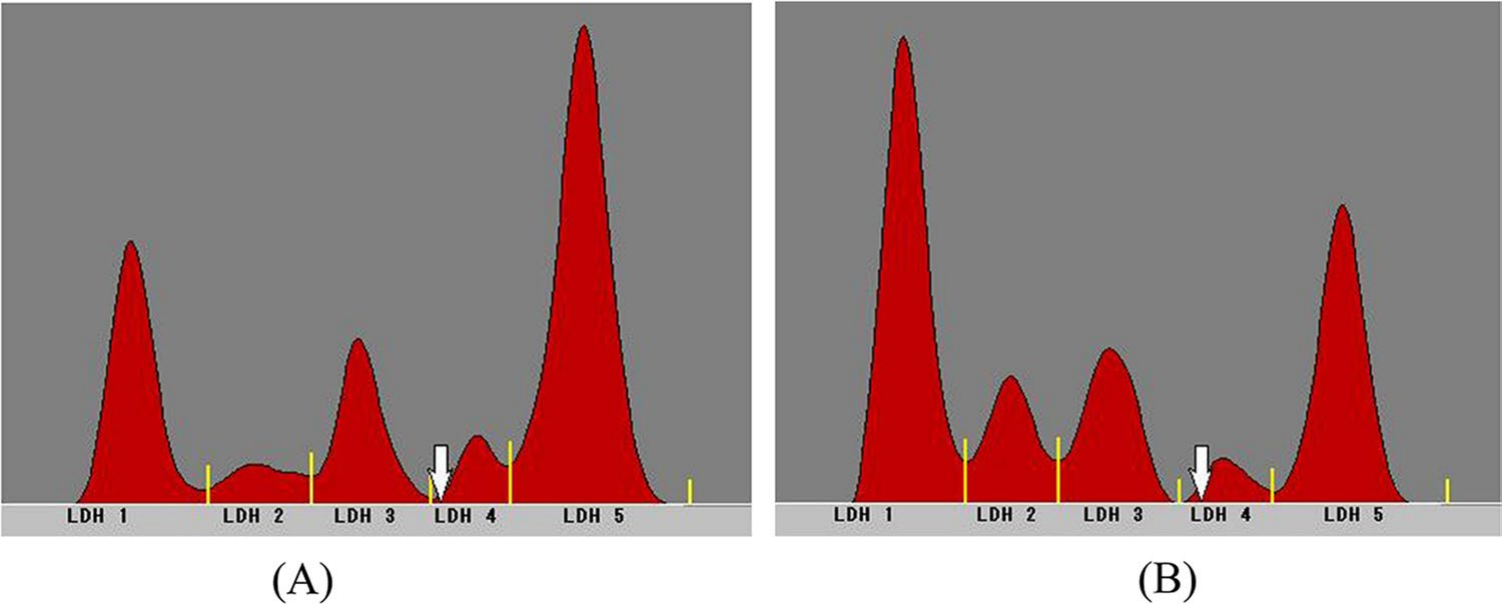
Representative electrophoretic patterns of LDH isozymes in the plasma of a dog with IVDD/CVDH before (**A**) and after (**B**) acupuncture treatment. Plasma LDH isozyme patterns were detected by biphasic agarose gel electrophoresis utilizing commercial Quick LD gels (Helena Laboratories, Saitama, Japan). The LDH fraction was assessed and analyzed using Quick Scan (Helena Laboratories, Saitama)

Representative electrophoretic patterns of plasma LDH isozymes in a dog with IVDD were shown in Fig. 1. After acupuncture treatment, the LDH 5 fraction decreased from 65.07% to 38.83%, while the LDH1fraction increased from 17.40% to 31.84% in 10 dogs with IVDD that showed improvement (Table 2). Six dogs that did not show improvement did not exhibit significant changes in isozyme proportions.

## Discussion

IVDD /CVDH is often observed in dogs, and its prevalence increases with aging (Jeffery et al. 2013). Recently, Chinese traditional medicine has been applied to treat IVDD (Roynard et al. 2018; Wright 2019). Acupuncture therapy can effectively alleviate oxidative stress in various diseases (Su et al. 2020, Zeng et al. 2014).

The LDH5 fraction is generally predominant in canine plasma (Washizu et al. 2002). However, after acupuncture treatments, dogs with IVDD/CVDH that showed improvement had decreased plasma pyruvate concentrations and increased LDH1 fractions. This finding suggests that acupuncture treatments may change the local redox potential (Zeng et al. 2014), and increase nicotinamide adenine dinucleotide (NAD) levels resulting in the conversion of lactate to pyruvate and activation of the citric acid cycle. LDH 1, which has a lower Km value for pyruvate, usually functions under aerobic condition. The plasma M/L ratio is indicators of oxidative metabolism; specifically, an increased M/L ratio may suggest increased ATP production via the citric acid cycle (Li et al. 2012).

We typically administered electroacupuncture to animals with sever paralysis, since this method applies a constant stimulus not only to the acupuncture points but also to the surrounding muscles. The animals that receive electroacupuncture showed continuous muscle contraction for 20 min. Thus, the stimulation due to electroacupuncture appears stronger than that of needle acupuncture. If an animal seemed to dislike the electroacupuncture method, we applied the needle acupuncture method. After electroacupuncture treatment, the pain experienced by the animals appeared to be alleviated, although we did not evaluate the severity of pain in this study.

The limitations in this study are as follows.

1. This study was a pilot study. We did not include a sham group, or a control group.
2. The acupuncture effect may be transient. A long-term follow-up of acupuncture effects is needed.
3. The acupuncture points were difficult to confirm. However, acupuncture treatments were administered by an experienced clinician.
4. The mechanism underlying differences in the effects of electroacupuncture and needle acupuncture methods remains unclear.

Additionally, further studies with larger sample sizes should be performed to confirm the present findings.

## Data Availability

The datasets generated during the present study are available from the corresponding author on request.

## Abbreviations

ATP: Adenosine triphosphate
CD: Chondrodystrophic
CVDH: Cervical disk herniation
IVDD: Intervertebral disk disease
LDH: Lactate dehydrogenase
MDH: Malate dehydrogenase
M/L ratio: Malate dehydrogenase/lactate dehydrogenase ratio
NCD: Nonchondrodystrophic
